# Risk factors for postoperative delirium in elderly patients undergoing heart valve surgery with cardiopulmonary bypass

**DOI:** 10.1186/s13019-024-02568-3

**Published:** 2024-02-22

**Authors:** Na Chen, Yun-chang Mo, Min Xu, Si-si Chen, Wei Gao, Qiong Zheng, Jue Wang, Xiao-chu Wang, Jun-lu Wang

**Affiliations:** 1 Department of Surgical Anesthesiology, The First Affiliated Hospital of Wenzhou Medical University, Wenzhou, Zhejiang 325000 China; 2 Department of Geriatric Medicine, Wenzhou Central Hospital, Wenzhou, Zhejiang 325000 China; 3 Department of Cardiac Surgery, The First Affiliated Hospital of Wenzhou Medical University, Wenzhou, Zhejiang 325000 China

**Keywords:** Heart valve surgery, Cardiopulmonary bypass, Cerebral oxygen saturation, Postoperative delirium, Elderly

## Abstract

**Background:**

The aim of this study was to identify the risk factors for postoperative delirium (POD) in elderly patients undergoing heart valve surgery with cardiopulmonary bypass (CPB).

**Methods:**

Elderly patients undergoing elective heart valve surgery with CPB in The First Affiliated Hospital of Wenzhou Medical University between March 2022 and March 2023 were selected for this investigation. They were divided into a POD group and a non-POD group. Their baseline information was collected and recorded, and the patients were subjected to neurocognitive function assessment using the Mini-Mental State Examination and the Montreal Cognitive Assessment scales before surgery. We also recorded their intraoperative indicators such as duration of surgery, duration of CPB, duration of aortic cross-clamp, blood transfusion, and postoperative indicators such as duration of mechanical ventilation, postoperative 24-hour drainage volume, and pain score. Regional cerebral oxygen saturation was monitored intraoperatively by near-infrared spectroscopy based INVOS5100C Regional Oximeter. Patients were assessed for the occurrence of POD using Confusion Assessment Method for the Intensive Care Unit, and logistic regression analysis of risk factors for POD was performed.

**Results:**

The study finally included 132 patients, with 47 patients in the POD group and 85 ones in the non-POD group. There were no significant differences in baseline information and preoperative indicators between the two groups. However, marked differences were identified in duration of surgery, duration of CPB, duration of aortic cross-clamp, duration of postoperative mechanical ventilation, postoperative length of stay in cardiac intensive care unit, postoperative length of hospital stay, intraoperative blood transfusion, postoperative pain score, and postoperative 24-hour drainage volume between the two groups (*p* < 0.05). Additionally, the two groups had significant differences in rScO_2_ at each intraoperative time point and in the difference of rScO_2_ from baseline at each intraoperative time point (*p* < 0.05). Multivariate logistic regression analysis showed that duration of surgery > 285 min (OR, 1.021 [95% CI, 1.008–1.035]; *p* = 0.002), duration of postoperative mechanical ventilation > 23.5 h (OR, 6.210 [95% CI, 1.619–23.815]; *p* = 0.008), and postoperative CCU stay > 3.5 d (OR, 3.927 [95% CI, 1.046–14.735]; *p* = 0.043) were independent risk factors of the occurrence of POD while change of rScO_2_ at T_1_>50.5 (OR, 0.832 [95% CI 0.736–0.941]; *p* = 0.003) was a protective factor for POD.

**Conclusion:**

Duration of surgery duration of postoperative mechanical ventilation and postoperative CCU stay are risk factors for POD while change of rScO_2_ at T_1_ is a protective factor for POD in elderly patients undergoing heart valve surgery with CPB.

## Background

Valve heart disease (VHD) is the most common structural heart disease, referring to anatomical and functional abnormalities of the heart valves or perivalvular tissue caused by rheumatic conditions, degenerative conditions, infections, or congenital malformations [1]. As the population ages, the incidence of VHD is progressively rising. According to the report from American Heart Association, the incidence of moderate to severe VHD can reach 1.8%. More significantly, the incidence increases with patient age, with 4.4% occurring in the population under 65 years of age and 11.7% in the population over 75 years of age [2]. Heart valve replacement or valvuloplasty under cardiopulmonary bypass (CPB) is one of the most common treatments for VHD [3]. However, different postoperative complications can arise as a result of complex surgery, old age, and other complicating factors [4]. Active precautions need to be taken since postoperative complications after heart valve surgery under CPB affect the prognosis and quality of life of patients.

Recent years have seen amazing advancements in cardiac surgery techniques and perioperative management. As a result, in addition to death and serious postoperative complications such vital organ failure, minor or even imperceptible complications, like postoperative delirium (POD), have received increasing attention. POD is a common postoperative complication after general anesthesia and surgery. As an indicator of acute cerebral dysfunction, POD is accompanied by concentration difficulty, disturbed consciousness, declined cognitive ability, or perceptual disorders, especially in the elderly. POD usually develops quickly after surgery (between a few hours and three days) and the condition fluctuates with time [5, 6]. Studies have shown that the incidence of POD in patients undergoing cardiac surgery ranges from 4.1 to 54.9%, with elderly patients experiencing a higher incidence of over 80% [7, 8]. Additionally, POD may lead to long-term postoperative cognitive dysfunction (POCD), necessitating a referral to a rehabilitation facility for continued treatment after discharge. Even worse, POD may increase in-hospital mortality and long-term post-discharge mortality [9]. Melatonin may be effective in lessening the severity of POD after cardiac surgery [10]. However, it is more important to take measures to prevent the occurrence of POD.

The occurrence of POD will increase the difficulty of postoperative care and the workload of nurses. Clinical and nursing staff have begun to realize the importance of delirium management and pay attention to the assessment, prevention and treatment of delirium in recent years [11]. A recent meta-analysis showed that regional cerebral oxygen desaturations were significantly associated with neurological complications after cardiac surgery, and that monitoring cerebral oximetry during cardiac surgery could help lower the incidence of postoperative cognitive dysfunction or stroke [12]. However, this conclusion was drawn based on data from the general population; very few research concentrated on the elderly patients. In this study, we analyzed the risk factors associated with the occurrence of POD in elderly patients undergoing heart valve surgery with CPB. It is anticipated that our findings would provide some theoretical basis for the prevention of POD in clinical practice.

## Study subjects and methods

### Study subjects

This study was an observational case-control study. Elderly patients who underwent elective heart valve surgery under CPB at The First Affiliated Hospital of Wenzhou Medical University from March 2022 to March 2023 were selected and divided into a POD group and a non-POD group according to whether or not they developed POD. Confusion Assessment Method for the Intensive Care Unit (CAM-ICU) was used for screening and diagnosis of POD. Inclusion criteria were as follows: (1) patients receiving elective cardiac valve surgery under CPB; (2) patients aged ≥ 65 years old; (3) patients or their family member who agreed to sign an informed consent. Exclusion criteria were as follows: (1) patients with hepatic insufficiency or chronic obstructive pulmonary disease; (2) patients with a history of central nervous system diseases such as delirium, cerebrovascular accident, and dementia or a history of psychosis; (3) patients receiving emergency surgery; (4) patients with carotid artery stenosis ≥ 60%; (5) patients receiving concurrent cardiac coronary artery bypass surgery or operation on great vessels; (6) patients who refused to be followed up. This study complied with the *Declaration of Helsinki* and gained approval from The First Affiliated Hospital of Wenzhou Medical University Ethics Committee (ethics number: KY2021-130). Informed consent forms were signed by the included patients and their families.

### Observation indicators

Indicators recorded preoperatively included sex, age, body mass index (BMI), body surface area, and European system for cardiac operative risk evaluation (EuroSCORE), education level (years), medical history (hypertension, diabetes, atrial fibrillation, cerebral infarction), history of adverse habits (smoking, alcohol), Mini-mental state examination (MMSE) score, Montreal Cognitive Assessment (MoCA) score, and history of other surgical procedures. The recorded perioperative blood parameters included hemoglobin, platelets, serum albumin, urea nitrogen, serum creatinine, estimated glomerular filtration rate, serum calcium, total cholesterol, creatine kinase, creatine kinase-MB (CK-MB), and left ventricular ejection fraction (LVEF). We also recorded intraoperative conditions including type of surgery, duration of surgery, duration of CPB, duration of aortic cross-clamp, and blood transfusion, and postoperative conditions including duration of postoperative mechanical ventilation, postoperative 24-hour drainage volume, postoperative pain score, postoperative cardiac ICU (CCU) stay, and postoperative hospital stay.

### Neurocognitive function assessment

MMSE and MoCA scales were responsible for preoperative assessment of neurocognitive function. Given their high sensitivity and specificity, the two scales are most widely used for the screening test of cognitive function in China and even in the world [13–15]. The test based on MMSE and MoCA aimed to exclude patients with preoperative cognitive dysfunction. The test was completed by one investigator who was instructed by a neurologist with relevant experience and was trained in more than 10 patients during the pre-experimental phase. In consideration of the patients’ biological clock and preoperative preparations, the test was conducted in the wards between 18:00 pm–20:00 pm the night before surgery. The ward environment was kept quiet and free of disturbances, and the help and reminders of patients’ families were avoided during the test. Patients who were unable to communicate verbally before surgery or who were unable to continue the test due to fatigue or other discomfort during the test were excluded from the study. Since the MMSE and MoCA scores were closely related to the level of education, the assessment scores were adjusted according to education level. The MMSE criteria for each education level were as follows: 17 points for the illiterate group (no education), 20 points for the primary school group (≤ 6 years of education), 22 points for the secondary school group, and 24 points for the university group; patients with corresponding education level who scored below these criteria were excluded from the study. One point was added to the MoCA test results for patients with < 12 years of education, and 2 points were added for patients with < 9 years of education, so as to correct the bias of education level [16].

### Perioperative management

After the patient was admitted to the operating room, routine anesthesia for cardiac surgery was performed, with the monitoring of electrocardiogram, pulse oxygen saturation, and bispectral index. Additionally, invasive arterial pressure was monitored through the cannulation of left radial artery. After induction of anesthesia with face mask (oxygen flow rate: 3 L/min), rapid sequence intubation was conducted and a transesophageal echocardiographic probe was placed through the mouth. Etomidate (0.3 mg/kg), midazolam (0.03 mg/kg), sufentanil (1–5 µg/kg), and cis-atracurium (0.1–0.2 mg/kg) were administered through peripheral veins for the induction of anesthesia, and meanwhile, dexmedetomidine (0.5 µg/kg/h) was given continuously. 0.6–0.7 MAC sevoflurane was inhaled or 0.02% norepinephrine was injected as needed for depth of anesthesia or hemodynamics adjustment. During maintenance of anesthesia, end-tidal carbon dioxide was maintained at 30–40 mmHg by adjusting the minute ventilation.

Before performing intubation, 4 mg/kg of heparin sodium was given intravenously, and 30 mg of heparin sodium was added to the CPB prime solution to wet the tube. After 5 min of intravenous heparinization, activated clotting time (ACT) was monitored, with ACT > 480 s allowing the start of intubation and extracorporeal circulation. During extracorporeal circulation, ACT was recorded every 60–90 min and maintained at ≥ 480 s; 50–100 mg of heparin sodium was added when ACT < 480 s. For nonpulsatile CPB with mild hypothermia, crystalloid St.Tomas cardioplegic solution was prepared and perfused into the body at a ratio of 1:4 with oxygenated blood, with a total amount of 20 ml/kg for the first perfusion, and with the dose of body surface area (BSA)×2.0-2.5 L/min to keep the O_2_ flow within 2-2.5 L for maintaining perfusion. The body temperature was monitored by dual channels, and nasopharyngeal and bladder temperatures were both maintained at 32–36 ℃. With bladder temperature as reference, the difference between nasopharyngeal temperature and bladder temperature was maintained at < 2 ℃ during rewarming. CPB flow rate was kept at 2.2–2.8 L/(min·m^2^). Invasive mean arterial pressure (MAP) of 50–80 mmHg, arterial partial pressure of oxygen of 150–250 mmHg and partial pressure of carbon dioxide of 35–45 mmHg were maintained throughout CPB.

### Regional cerebral oxygen saturation monitoring

The device used to monitor regional cerebral oxygen saturation (rScO_2_) in this study was the near-infrared spectroscopy (NIRS) based INVOS5100C Regional Oximeter (Medtronic, USA). The patient was admitted to the operating room in a supine position on the operating table. Two self-adhesive NIRS sensors were fixed to the forehead (1–2 cm above the eyebrows) after wiping the forehead with an alcohol cotton ball. Then the patient inhaled air in the operating room in a conscious and quiet state, and rScO_2_ value was observed 1 min later until the value stabilized; the stable rScO_2_ value was read as the baseline value (T_0_). Monitoring of rScO_2_ was stopped at the end of surgery. Four intraoperative times points were selected: start of anesthesia (T_1_), start of CPB (T_2_), start of rewarming (T_3_), and end of CPB (T_4_). The values of rScO_2_ at T_1_, T_2_, T_3_, and T_4_ were recorded. Then the changes of rScO_2_ at each time point compare with rScO_2_ at T_0_ (change of rScO_2_ at T_1_ = rScO_2_ at T_1_ - rScO_2_ at T_0_, change of rScO_2_ at T_1_ = rScO_2_ at T_0_ - rScO_2_ at T_2_, change of rScO_2_ at T_3_ = rScO_2_ at T_0_ - rScO_2_ at T_3_, and change of rScO_2_ at T_4_ = rScO_2_ at T_4_ - rScO_2_ at T_0_) were calculated. When rScO_2_ falls below absolute values of 50% or less than 20% of the baseline, doctors would directly or indirectly increase cerebral oxygen values by measures such as increasing aortic perfusion flow or increasing oxygen flow or drugs, while the extracorporeal circulation physician needs to take into account the maintenance of vital signs such as creation of a mean arterial pressure of 50–80 mmHg, maintenance of arterial partial pressures of oxygen of 150–250 mmHg and carbon dioxide partial pressures of 35–45 mmHg in a relatively stable manner.

### Postoperative delirium assessment

The patient was transferred to the CCU immediately after cardiac surgery. The patient was assessed for POD by a CCU nurse specialist using CAM-ICU. The CAM-ICU defines POD based on four diagnostic features: acute change or fluctuating course of mental status, inattention, disorganized thinking, and altered level of consciousness [17]. Once a patient was admitted to the CCU, POD assessment was initiated using the CAM-ICU and the duration of delirium was recorded. Each patient was scored every 6–8 h before transfer to the general ward or 7 days after surgery. The evaluator was not a member of the study team and did not have access to any other information of this research.

### Statistical analysis

SPSS 25.0 was used for statistical analysis. For continuous data, the Kolmogorove Smirnov test was first applied to assess whether the data were normally distributed. If the data of both groups met normality and the variances between the them were equal, the t-test was responsible for comparison between groups and data were expressed as mean ± standard deviation (SD); otherwise the nonparametric Wilcoxon rank sum test was considered. For categorical data, the chi-square test was responsible for comparison between groups and data were expressed as number of cases (percentage) [n (%)]. Additionally, repeated measures analysis of variance was considered for determining whether there were statistical differences of same parameters between the groups and time points, respectively. The interaction terms between groups and time points were analyzed to explore whether there were differences in change trends between the groups. If the interaction terms were statistically significant, it suggested that the change trend over time may differ between groups. Risk factors for POD were analyzed by binary logistic regression. First univariate logistic regression was used for the initial screening of risk factors, and then the factors with *p* < 0.05 in univariate logistic regression were included in multivariate logistic regression. A *p* value < 0.05 was considered statistically significant in multivariate logistic regression.

## Study results

### General information of patients

One hundred and fifty-five patients were included in the study based on the inclusion and exclusion criteria. Among them, there were 8 cases with intraoperative changes of surgical methods (combined with coronary artery bypass surgery or operation on great vessels) and 15 cases with missing intraoperative data. Therefore, 132 cases with complete data were finally statistically analyzed (POD group: *n* = 47; non-POD group: *n* = 85). Baseline data of patients are shown in Table 1. The POD and non-POD patients had no significant differences in terms of age (70 ± 5 vs. 70 ± 4), sex (M/F: 24/23 vs. 43/42), education level (illiterate/primary/secondary/university: 16/22/5/4 vs. 28/36/15/6), BMI (21.81 ± 3.08 vs. 22.29 ± 2.87), body surface area (1.86 ± 1.97 vs. 1.61 ± 0.14), diabetes mellitus (19.15% vs. 20%), hypertension (38.18% vs. 52.90%), history of smoking (40.43% vs. 47.06), history of alcohol consumption (38.30% vs. 43.53%), history of atrial fibrillation (8.51% vs. 7.06%), history of cerebral infarction (8.51% vs. 4.71%), history of other surgeries (14.89% vs. 14.12%), type of surgery (single-valve/multivalve: 20/27 vs. 50/35), dosage of midazolam (1.83 ± 0.32 vs. 1.77 ± 0.30), mean arterial pressure (76 ± 5.35 vs. 76 ± 6.38), and mean temperature (35.52 ± 0.19 vs. 35.32 ± 0.23).

**Table 1:** Clinical baseline information of patients in both groups

General information	POD group (*n* = 47)	Non-POD group (*n* = 85)	*p*-value
Age (mean ± SD) (years)	70 ± 5	70 ± 4	0.364
Sex n (%)			0.958
Male	24 (51.1)	43 (50.5)	
Female	23 (48.9)	42 (49.5)	
Educational level n (%)			0.750
Illiterate group = 0	16 (34.0)	28 (32.9)	
0 < Primary school group ≤ 5	22 (46.7)	36 (42.2)	
5 < Secondary school group ≤ 11	5 (10.5)	15 (17.5)	
University group ≥ 12	4 (8.4)	6 (6.9)	
BMI (mean ± SD) (kg/m^2^)	21.81 ± 3.08	22.29 ± 2.87	0.565
BSA (mean ± SD) (m^2^)	1.86 ± 1.97	1.61 ± 0.14	0.966
Medical history n (%)			
Diabetes	9 (19.15)	17 (20.00)	0.906
Hypertension	21 (44.68)	45 (52.94)	0.363
History of smoking	19 (40.43)	40 (47.06)	0.463
History of alcohol consumption	18 (38.30)	37 (43.53)	0.559
Atrial fibrillation	4 (8.51)	6 (7.06)	0.744
Cerebral infarction	4 (8.51)	4 (4.71)	0.454
History of other surgical procedures	7 (14.89)	12(14.12)	0.903
Type of surgery n (%)			0.073
Single-valve	20 (42.55)	50 (58.82)	
Multivalve	27 (57.45)	35 (41.18)	
Dosage of midazolam (mg)	1.83 ± 0.32	1.77 ± 0.30	0.244
Mean arterial pressure (mmHg)	76 ± 5.35	76 ± 6.38	0.881
Mean temperature (℃)	35.52 ± 0.19	35.32 ± 0.23	0.738

*Note* POD, postoperative delirium; BMI, body mass index; BSA, body surface area. t-test was used for comparison of age, BMI, and BSA between groups. The mean arterial pressure was the mean blood pressure at preoperative, after general anesthesia, after CPB, at the end of arterial pressure, and at the end of surgery. Mean temperature was the mean temperature at preoperative, after CPB, minimum temperature at CPB, at the end of CPB, and at the end of surgery

### Preoperative indicators in the POD and non-POD groups

The preoperative indicators of the patients are shown in Table 2. The two groups showed no significant differences in preoperative blood parameters, including hemoglobin (125.85 ± 16.98 vs. 126.35 ± 22.61), platelets (200.69 ± 76.59 vs. 196.70 ± 91.27), albumin (38.26 ± 4.88 vs. 39.76 ± 7.72), urea nitrogen (6.97 ± 3.95 vs. 6.03 ± 1.62), creatinine (80.78 ± 55.12 vs. 76.57 ± 24.57), estimated glomerular filtration rate (90.30 ± 22.61 vs. 133.40 ± 196.57), serum calcium (2.27 ± 0.14 vs. 2.23 ± 0.11), total cholesterol (125.85 ± 16.98 vs. 126.35 ± 22.61), creatine kinase (109.13 ± 181.93 vs. 144.74 ± 242.58), creatine kinase-MB (13.44 ± 24.62 vs. 13.75 ± 14.05), left ventricular ejection fraction (61.42 ± 8.15 vs. 64.80 ± 3.80) (*p* > 0.05). Additionally, no marked difference was identified between the two groups in terms of preoperative MMSE (21.6 ± 3.5 vs. 21.2 ± 3.1), preoperative MoCA (20.2 ± 3.1 vs. 20.9 ± 2.7), and EuroSCORE (3.26 ± 0.14 vs. 3.12 ± 0.10) (*p* > 0.05).

**Table 2:** Comparison of preoperative indicators between the two groups

Preoperative indicators (mean ± SD)	POD group (*n* = 47)	Non-POD group (*n* = 85)	*p*-value
Hemoglobin (g/L)	125.85 ± 16.98	126.35 ± 22.61	0.279
Platelets (*10^9^ /L)	200.69 ± 76.59	196.70 ± 91.27	0.147
Albumin (g/L)	38.26 ± 4.88	39.76 ± 7.72	0.185
urea nitrogen (mmol/L)	6.97 ± 3.95	6.03 ± 1.62	0.765
Creatinine (umol/L)	80.78 ± 55.12	76.57 ± 24.57	0.885
eGFR (ml/Min)	90.30 ± 22.61	133.40 ± 196.57	0.153
Serum calcium (mmol/L)	2.27 ± 0.14	2.23 ± 0.11	0.353
Total cholesterol (mmmol/L)	125.85 ± 16.98	126.35 ± 22.61	0.613
Creatine kinase (U/L)	109.13 ± 181.93	144.74 ± 242.58	0.414
CK-MB (U/L)	13.44 ± 24.62	13.75 ± 14.05	0.576
LVEF (%)	61.42 ± 8.15	64.80 ± 3.80	0.833
Preoperative MMSE	21. 6 ± 3.5	21.2 ± 3.1	0.805
Preoperative MoCA	20.2 ± 3.1	20.9 ± 2.7	0.400
EuroSCORE	3.26 ± 0.14	3.12 ± 0.10	0.424

*Note* POD, postoperative delirium; eGFR, estimated glomerular filtration rate; CK-MB, creatine kinase-MB; LVEF, left ventricular ejection fraction; MMSE, mini-mental state examination; MoCA, Montreal Cognitive Assessment; EuroSCORE, European system for cardiac operative risk evaluation

### Intraoperative regional cerebral oxygen saturation in the POD and non-POD groups

There was no discernible difference in preoperative baseline values of rScO_2_ (T_0_) between the two groups (*p* > 0.05). rScO_2_ values of POD patients and non-POD patients were recorded continuously during surgery; rScO_2_ at all intraoperative time points in the POD group were smaller than those in the non-POD group. Repeated measures analysis of variance confirmed that the differences were all considered statistically significant (*p* < 0.01) (Table 3). Meanwhile, the difference between rScO_2_ at each intraoperative time point and rScO_2_ at baseline in the two groups was subjected to *t*-test. Compared to the non-POD group, the rScO_2_ values in the POD group showed a smaller increase and a larger decrease from baseline (*p* < 0.01) (Table 3).

**Table 3:** Comparison of intraoperative regional cerebral oxygen saturation between the two groups

rScO_2_ value (mean ± SD)	POD group (*n* = 47)	Non-POD group (*n* = 85)	*p*-value
rScO_2_ at T_0_	58.2 ± 6.9	60.5 ± 7.7	0.089
rScO_2_ at T_1_	69.4 ± 8.7	74.9 ± 9.8	0.002
rScO_2_ at T_2_	46.5 ± 6.3	49.7 ± 7.8	0.018
rScO_2_ at T_3_	46.4 ± 6.1	49.4 ± 7.5	0.021
rScO_2_ at T_4_	62.8 ± 8.0	68.1 ± 9.4	0.001
Change of rScO_2_ at T_1_	11.0 ± 5.9	14.6 ± 5.3	0.002
Change of rScO_2_ at T_2_	12.0 ± 4.6	10.1 ± 4.4	0.024
Change of rScO_2_ at T_3_	13.1 ± 4.7	10.9 ± 4.8	0.013
Change of rScO_2_ at T_4_	5.9 ± 5.0	9.6 ± 5.6	0.001

*Note* POD, postoperative delirium; rScO_2_, regional cerebral oxygen saturation; rScO_2_ at T_0_, baseline value of rScO_2_; T_1_, start of anesthesia; T_2_, start of CPB; T_3_, start of rewarming; T_4_, end of CPB

### Intraoperative and postoperative indicators in the POD and non-POD groups

Significant differences were found in duration of surgery (295.8 ± 23.4 vs. 274.7 ± 19.9), duration of CPB time (155.9 ± 17.9 vs. 142.9 ± 16.1), duration of aortic cross-clamp (132.1 ± 57.0 vs. 100.8 ± 38.8), intraoperative blood transfusion (78.8% vs. 48.2%), duration of postoperative mechanical ventilation (26.7 ± 22.5 vs. 18.5 ± 8.9), postoperative CCU stay (5.5 ± 2.1 vs. 4.0 ± 1.2), postoperative hospital stay (26.6 ± 9.9 vs. 19.4 ± 5.6), postoperative pain score (1.4 ± 0.2 vs. 0.9 ± 0.1), and postoperative 24-hour drainage volume (264.6 ± 6.2 vs. 218.1 ± 3.7) between the POD and non-POD groups (*p* < 0.05) (Table 4).

**Table 4:** Comparison of intraoperative and postoperative indicators between the two groups

Intraoperative and postoperative indicators (mean ± SD)	POD group (*n* = 47)	Non-POD group (*n* = 85)	*p*-value
Duration of surgery (min)	295.8 ± 23.4	274.7 ± 19.9	< 0.001
Duration of CPB (min)	155.9 ± 17.9	142.9 ± 16.1	< 0.001
Duration of aortic cross-clamp (min)	132.1 ± 57.0	100.8 ± 38.8	< 0.001
Duration of postoperative mechanical ventilation (h)	26.7 ± 22.5	18.5 ± 8.9	< 0.001
Postoperative CCU stay (d)	5.5 ± 2.1	4.0 ± 1.2	< 0.001
Postoperative hospital stay (d)	26.6 ± 9.9	19.4 ± 5.6	0.001
Postoperative pain score	1.4 ± 0.2	0.9 ± 0.1	0.034
Postoperative 24-hour drainage volume (ml)	264.6 ± 6.2	218.1 ± 3.7	0.001
Intraoperative blood transfusion n (%)			0.001
Yes	37 (78.80)	41 (48.20)	
No	10 (21.20)	44 (51.80)	

*Note* POD, postoperative delirium; CPB, cardiopulmonary bypass; CCU, cardiac intensive care unit

### Risk factor analysis of POD

Risk factors for POD were analyzed using binary logistic regression. First univariate logistic regression analysis showed that duration of surgery > 285 min (OR, 1.015 [95% CI, 1.005–1.025]; *p* = 0.003), duration of CPB > 156 min (OR, 1.018 [95% CI, 1.010–1.027]; *p* = 0.001), duration of aortic cross-clamp > 133.5 min (OR, 1.020 [95% CI, 1.011–1.029]; *p* = 0.001), duration of postoperative mechanical ventilation > 23.5 h (OR, 1.053 [95% CI, 1.025–1.082]; *p* = 0.001), postoperative CCU stay > 3.5 d (OR, 1.486 [95% CI, 1.209–1.827]; *p* = 0.001), Postoperative hospital stay > 24.5 d (OR, 1.088 [95% CI, 1.033–1.146]; *p* = 0.001), Postoperative pain score > 1.5 (OR, 1.333 [95% CI, 1.003–1.772]; *p* = 0.048), Change of rScO_2_ at T_1_ > 50.5 (OR, 0.870 [95% CI, 0.804–0.940]; *p* = 0.001), Change of rScO_2_ at T_2_ > 39.25 (OR, 1.097 [95% CI, 1.011–1.189]; *p* = 0.026), Change of rScO_2_ at T_3_ > 37.5 (OR, 1.099 [95% CI, 1.018–1.186]; *p* = 0.015) and change of rScO_2_ at T_4_ > 51 (OR, 0.865 [95% CI, 0.795–0.942]; *p* = 0.001) were possible predictors of the occurrence of POD. The above indicators were included in a multivariate regression model. Multivariate regression analysis demonstrated that duration of surgery (OR, 1.021 [95% CI, 1.008–1.035]; *p* = 0.002), duration of postoperative mechanical ventilation > 23.5 h (OR, 6.210 [95% CI, 1.619–23.815]; *p* = 0.008), and postoperative CCU stay > 3.5 d (OR, 3.927 [95% CI, 1.046–14.735]; *p* = 0.043) were independent risk factors of the occurrence of POD while change of rScO_2_ at T_1_ > 50.5 (OR, 0.832 [95% CI 0.736–0.941]; *p* = 0.003) was a protective factor of POD (Table 5).

**Table 5:** Univariate logistic regression analysis of risk factors for occurrence of POD

Variables	Univariate analysis				Multivariate analysis			
	*p*-value	OR	95% CI of OR		*p*-value	OR	95% CI of OR	
			Upper	Lower			Upper	Lower
Duration of surgery > 285 min	0.003	1.015	1.005	1.025	0.002	1.021	1.008	1.035
Duration of CPB > 156 min	0.001	1.018	1.010	1.027	0.236	0.983	0.956	1.011
Duration of aortic cross-clamp > 133.5 min	0.001	1.020	1.011	1.029	0.432	1.011	0.984	1.039
Duration of postoperative mechanical ventilation > 23.5 h	0.001	1.053	1.025	1.082	0.008	6.210	1.619	23.815
Postoperative CCU stay > 3.5 d	0.001	1.486	1.209	1.827	0.043	3.927	1.046	14.735
Postoperative hospital stay > 24.5 d	0.001	1.088	1.033	1.146	0.775	1.207	0.333	4.371
Postoperative pain score > 1.5	0.048	1.333	1.003	1.772	0.050	3.309	1.001	10.942
Intraoperative blood transfusion	0.374	1.577	0.578	4.298	0.861	1.120	0.318	3.947
Change of rScO_2_ at T_1_ > 50.5	0.001	0.870	0.804	0.940	0.003	0.832	0.736	0.941
Change of rScO_2_ at T_2_ > 39.25	0.026	1.097	1.011	1.189	0.966	1.005	0.797	1.267
Change of rScO_2_ at T_3_ > 37.5	0.015	1.099	1.018	1.186	0.707	1.044	0.834	1.306
Change of rScO_2_ at T_4 _> 51	0.001	0.865	0.795	0.942	0.522	0.961	0.850	1.086

*Note* POD, postoperative delirium; CPB, cardiopulmonary bypass; CCU, cardiac intensive care unit; rScO_2_, regional cerebral oxygen saturation; rScO_2_ at T_0_, baseline value of rScO_2_; T_1_, start of anesthesia; T_2_, start of CPB; T_3_, start of rewarming; T_4_, end of CPB

## Discussion

The nomenclature of postoperative neurocognitive disorders is consistent with the Diagnostic and Statistical Manual of Mental Disorders, fifth edition (DSM-5), as per the latest published recommendations for the nomenclature of cognitive change associated with anaesthesia and surgery. In DSM-5, postoperative neurocognitive disorder is divided into two phases: the acute phase (from hours after surgery to hospital discharge) called POD and the chronic phase (30 days to 5 years after surgery or death) called POCD [18]. Therefore, the main observation period of POD in this study was from the transfer to the CCU to discharge. The uniform observation period and definition of POD make the study results more comparable and facilitate the interpretation of the study results. A total of 132 elderly patients undergoing heart valve surgery under CPB were included in this study, and the incidence of POD in CCU was 35.6%. MMSE and MoCA scales were used in this study to exclude patients with preoperative cognitive dysfunction. The occurrence of POD was significantly associated with duration of surgery, duration of CPB, duration of aortic cross-clamp, duration of postoperative mechanical ventilation, postoperative CCU stay, postoperative hospital stay, intraoperative blood transfusion, postoperative pain score, and postoperative 24-hour drainage volume. POD patients and non-POD patients showed significant differences in T_1_–T_4_ and change of rScO_2_ at T_1_-T_4_. Further duration of surgery, duration of postoperative mechanical ventilation and postoperative CCU stay were confirmed as independent risk factors for POD and change of rScO_2_ at T_1_ was a protective factor for POD.

Previous studies have different definitions of POD and different ranges of low rScO_2_. For example, in a randomized controlled trial by Lei et al., a lower incidence of POD was found in patients receiving a ScO_2_ monitoring and an intervention for intraoperative rScO_2_ below 75% of the baseline value than the controls with no rScO_2_ monitoring and no intervention for rScO_2_ [19]. Schoen et al. conducted an observational study of 231 patients, and the results showed a 4-fold increase in the rate of POD in patients with rScO_2_ ≤ 50% compared to those with rScO_2_ > 50%. They concluded that preoperative rScO_2_ < 59.5% was a high risk factor for POD after cardiac surgery [20]. In the study of Hong et al., patients undergoing elective valvular heart surgery under CPB were assessed for neurocognitive function using MMSE 1 day before and 7 days after surgery. As a result, they observed that no association existed between the decrease in intraoperative rScO_2_ and occurrence of POCD, and that patients with reduced ScO_2_ had a much longer postoperative hospital stay [21]. The difference in preoperative rScO_2_ baseline values between the two groups was not statistically significant in our study, but during surgery, the absolute value of lowest rScO_2_ was around 45% in POD patients and above 50% in non-POD patients. It is clear that our findings are consistent with other previous studies [22, 23].

Intraoperative rScO_2_ values obtained from NIRS-based monitoring continuously change with body temperature and surgical procedures at different time periods. Accordingly, trends in rScO_2_ over time must be taken into consideration in addition to a single intraoperative rScO_2_ value or preoperative baseline value in order to accurately predict the incidence of POD. A recent study demonstrated that significant changes in rScO_2_ during the rewarming phase of CPB were strongly associated with the postoperative neurocognitive recovery [24]. Jufar et al. reported that during CPB cardiac surgery, patients were vulnerable to neurological damage caused by microembolism and hypoperfusion and consequently developed impaired cerebral autoregulation [25]. Also, Joshi et al. showed that the impairment degree of cerebral autoregulation peaked during the rewarming phase of CPB [26]. Therefore, in this study, rScO_2_ T_0_–T_4_ were recorded, and based on these five values, the trend of intraoperative rScO_2_ and increase/decrease of intraoperative rScO_2_ were observed and analyzed. Further analysis in this study proved that rScO_2_ ΔT_1_ was a risk factor for POD, which supports the idea that the cooling phase of CPB may cause damage to cerebral blood flow autoregulation and put patients at risk of POD. Additionally, the decrease and increase of rScO_2_ in the POD group were significantly different from those in the non-POD group. The reason may be put down to that the cooling and rewarming phases of CPB impaired cerebral autoregulation, resulting in smaller magnitude of rScO_2_ increase and larger magnitude of rScO_2_ decrease. In contrast, rScO_2_ was monitored from 1 day before surgery to 72 h after surgery in Eertmans et al.‘s study, and no significant relationship was found between POD and intraoperative ScO_2_. However, there was a significant relationship between POD and a decrease in the relative or absolute value of postoperative ScO_2_ [27]. The limitation of this study is that each CAM-ICU assessment was performed at the end of an 8-hour shift, making it challenging to pinpoint when the decrease in rScO_2_ occurred-before, during, or after POD.

It is well known that short duration of surgery leads to relatively less anesthetic drug dosage, less surgical traum weaker and stress response in the brain. Conversely, lengthy surgeries are generally more traumatic, resulting in more blood loss, higher probability of blood transfusion, higher incidence of postoperative complications and higher difficulty of postoperative care. In our study, the POD group had longer duration of surgery and CPB, and aortic cross-clamp and postoperative mechanical ventilation and postoperative CCU stay than the non-POD group.First univariate logistic regression analysis showed that duration of surgery > 285 min and duration of CPB > 156 min and duration of aortic cross-clamp > 133.5 min and Change of rScO_2_ at T_1_ > 50.5 and Change of rScO_2_ at T_2_ > 39.25 and Change of rScO_2_ at T_3_ > 37.5 and change of rScO2 at T_4_ > 51 were possible predictors of the occurrence of POD (Table 5). For medical staff in the CCU, the occurrence of any one or several of the following situations during surgery in cardiac patients should be taken seriously as indicators that the patient may develop postoperative delirium. Furthermore, the multivariate logistic regression analysis showed that duration of surgery , duration of postoperative mechanical ventilation > 23.5 h (OR, 6.210 [95% CI, 1.619–23.815]; *p* = 0.008), and postoperative CCU stay > 3.5 d (OR, 3.927 [95% CI, 1.046–14.735]; *p* = 0.043) were independent risk factors of the occurrence of POD while change of rScO_2_ at T_1_ > 50.5 (OR, 0.832 [95% CI 0.736–0.941]; *p* = 0.003) was a protective factor of POD (Table 5). In order to minimize the risk of neurological complications following surgery, it is imperative that the CPB perfusionist has a thorough understanding of the surgical procedure, prevents patients from becoming too cold or too hot to shorten the rewarming phase, and avoids excessive temperature difference between the poikilothermia water tank and body temperature during the rewarming phase. Besides, medical staff are recommended to improve the efficiency of team communication, surgical skills and teamwork, so as to minimize the duration of surgery (< 285 min) and postoperative mechanical ventilation (< 23.5) h and postoperative CCU stay (< 3.5 d) and reduce POD incidence (Table 5). Some studies have shown that intraoperative blood transfusion, especially intraoperative plasma or platelet transfusion, is a high risk factor for POD [28, 29], similar to the results of our study. The CPB perfusionist can prepare autologous blood for transfusion before surgery or reduce the amount of CPB prime solution. Additionally, controlling intraoperative blood component loss can reduce the probability of intraoperative transfusion and thus lower the incidence of POD.

### Innovativeness and limitations

This study is innovative in the following ways. First, variability of rScO_2_ over time was taken into account for predicting the occurrence of POD requires in addition to a single intraoperative rScO_2_ value or preoperative baseline value. In this study, in addition to preoperative baseline, four intraoperative times points were selected: start of anesthesia (T_1_), start of CPB (T_2_), start of rewarming (T_3_), and end of CPB (T_4_). Second, NIRS-based INVOS5100C Regional Oximeter was used to automatically record rScO_2_ every 5 s and the data were stored offline in real time, allowing the extraction of rScO_2_ T_0_–T_4_. Finally, based on the above five values, the difference between the intraoperative ScO_2_ at each time point and the baseline value was determined. Therefore, it is possible to observe and analyze the trend of intraoperative rScO_2_, the magnitude of the increase and decrease of rScO_2_, and their correlation with POD.

However, this study still has several limitations. First, the change of postoperative neurocognitive function is a long-term postoperative manifestation, but this study lacks a long-term follow-up [30]. Second, this is a single-center observational study with small sample size, so our findings need to be further confirmed in a multicenter study with large sample size. Third, we included only patients with heart valve surgery under CPB, and future studies need to include patients with different surgical procedures or from specific age groups. Fourth, only one method (CAM-ICU) was used for the assessment of POD, and the three daily CAM-ICU assessments were performed at the end of an 8-hour shift. This assessment method and time may cause missed diagnosis of POD cases because delirium is a fluctuating syndrome. Fifth, we did not take into account intraoperative factors (e.g., hypo- or hyper-capnia, inflammatory factors) that may affect rScO_2_ and the risk of delirium. Finally, our ability to adjust confounding factors was limited, and some other variables that may influence the primary outcome were not analyzed in this study.

## Conclusion

Risk factors for POD in patients undergoing heart valve surgery under CPB include duration of surgery duration of postoperative mechanical ventilation and postoperative CCU stay. Besides, change of rScO_2_ at T_1_ was a protective factor for POD. Therefore, medical staff in CCU should pertinently manage POD for patients undergoing cardiac surgery depending on these risk factors.

## Data Availability

The data that support the findings of this study are available from the corresponding author upon reasonable request.
